# Ensuring proper and safe use of the cryotherapy machine

**Published:** 2014

**Authors:** Ismael Cordero

**Affiliations:** Clinical Engineer, Philadelphia, USA. ismaelcordero@me.com

**Figure:**
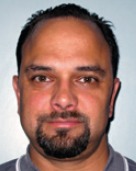
Ismael Cordero

Cryotherapy machines, also known as cryosurgery machines, continue to be widely used for surgical procedures of the eye such as retinal detachment repair, cataract extraction, glaucoma and so on.

Cryotherapy machines (Figure 1) control the release of a compressed gas, stored under high pressure in a cylinder. When the foot pedal is depressed, the gas is regulated to a lower working pressure and pumped to the metal tip of the cryoprobe, where it expands rapidly. This reduction in pressure cools the gas and the tip freezes very quickly. Release of the foot pedal causes warm low-pressure gas to flow through the probe for defrosting.

The main compressed gases used for cryotherapy are carbon dioxide (CO_2_) and nitrous oxide (N_2_O). Nitrous oxide is more effective for cryotherapy, but in many low-resource settings carbon dioxide is often much less expensive and more readily available.

The pressure gauge on the back of the machine (in the case of the model in Figure 1) indicates how much gas is inside the cylinder. The pressure gauge in the front indicates the regulated or ‘working’ pressure that is applied to the probe. This pressure can be adjusted using the knob on the front, according to the strength of cooling that is desired and the type of probe used, as per the manufacturer's recommendations. The higher the working pressure the greater the cooling.

Many probes have a thermocouple that measures the temperature at the tip and this measurement is displayed on the temperature gauge on the front of the machine. Most standard cryotherapy machines do not have to be connected to an electrical outlet but may require a small battery to power the temperature gauge.

## Avoiding exposure to N_2_O

The concentration of N_2_O in the room can reach several thousand parts per million during a cryosurgical procedure if the exhaust gas from the probe is not vented properly, and levels may remain elevated fora longtime. Exposure should be minimised to prevent the short-term behavioural and long-term reproductive health effects that can be caused by N_2_O.

Most modern N_2_O cryotherapy units are equipped with an exhaust. A length of plastic tubing available from the manufacturer can be connected directly to this port, with the other end connected to a discharge location outside the building. Remember:

**Figure 1:**
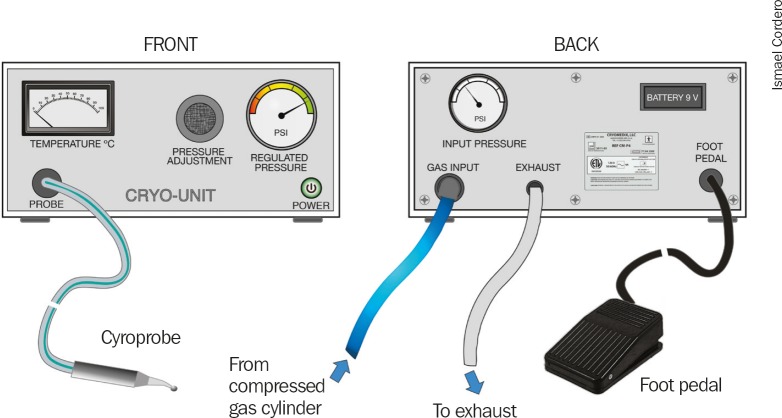

- Always discharge N_2_O to the outside, away from any air-intake ducts.
- Do not vent the N_2_O into a sink, drain trap, air recirculation duct, or the piped medical/surgical suction system.
- Consult with the manufacturer to determine which scavenging methods they recommend for their equipment.

## Before use

- Ensure that the gas cylinder is properly secured.
- Store the cylinders upright for a minimum of eight hours at ambient room temperature prior to use.
- Ensure that the gas cylinder is full, properly connected to the machine and that the cylinder valve is fully open.
- Ensure that the exhaust hose is connected and directed to a proper discharge location, as stated above.

The equipment should be tested immediately before use as follows:

- Use the foot pedal to release some gas with the probe tip immersed in water; a 2 cm ice ball should form at the tip.
- If the ice ball does not appear, then the machine is faulty or the gas cylinder is almost or completely empty.
- The freezing action should continue only so long as the foot pedal is depressed.

## During use

- Always vent N_2_O to the outside, away from any air-intake ducts.
- Do not constrict, kink, bend, lay objects on, or otherwise damage or restrict probe or exhaust lines.
- Do not attempt to insert or remove the probe at the probe jack when the cryosurgical machine is pressurised.

## After use

- Turn off the cylinder and depress the foot pedal to release all the gas in the tubing and probe. If this is not done, the tubing and probe can become damaged as the N_2_O leaves deposits inside the tubing.
- Wipe the machine with a damp cloth.
- Sterilise the tubing and probe according to the manufacturer's instructions.
- Wipe the foot pedal with a damp cloth and dry it before storing.
- Protect the machine with a plastic cover.
- Change the cylinder, if necessary, before the next operation.

## Basic troubleshooting

If the probe tip is not freezing sufficiently, this could be due to any off the following.

- **The regulator is not functioning,** which means there is not enough N_2_O coming through the machine. The regulator can be replaced, but an experienced technician should do this.
- **Leaking gas at the cylinder head.** Check that the hardened rubber O-ring at the junction of the cylinder and machine is there and in good condition.
- **An empty gas cylinder, or the pressure is too low in the cylinder.** Replace the cylinder.
- **Residual moisture in probe following sterilisation.** Re-purge the probe.
- **Probe blocked with particulate matter.** Replace the probe.
- **Exhaust hose is blocked or occluded.** Check the exhaust hose for blockages or occlusions and replace if necessary.

